# Assessing adverse effects and unspecific effects of transcutaneous spinal direct current stimulation (tsDCS)

**DOI:** 10.1162/IMAG.a.1292

**Published:** 2026-07-14

**Authors:** Hongyan Zhao, Ulrike Horn, Melanie Freund, Anna Bujanow, Christopher Gundlach, Gesa Hartwigsen, Falk Eippert

**Affiliations:** Max Planck Research Group Pain Perception, Max Planck Institute for Human Cognitive and Brain Sciences, Leipzig, Germany; Methods and Development Group Nuclear Magnetic Resonance, Max Planck Institute for Human Cognitive and Brain Sciences, Leipzig, Germany; Experimental Psychology and Methods, Wilhelm Wundt Institute for Psychology, Leipzig University, Leipzig, Germany; Cognitive and Biological Psychology, Wilhelm Wundt Institute for Psychology, Leipzig University, Leipzig, Germany; Lise Meitner Research Group Cognition and Plasticity, Max Planck Institute for Human Cognitive and Brain Sciences, Leipzig, Germany

**Keywords:** spinal cord, transcutaneous spinal direct current stimulation, adverse effects, unspecific effects, structured questionnaire, autonomic nervous system

## Abstract

Transcutaneous spinal direct current stimulation (tsDCS) is a relatively recent method for non-invasively modulating neural activity in the spinal cord. Despite its growing prominence, comprehensive studies addressing its potential adverse effects (AEs) and unspecific effects (UEs) are lacking. In this study, we conducted a systematic investigation of the potential AEs and UEs of tsDCS in healthy volunteers (N=20) who underwent a double-blind within-participant design, employing anodal, cathodal, and sham tsDCS of the thoracolumbar spinal cord. Our approach involved utilizing a newly developed structured questionnaire (to assess subjectively reported AEs) in combination with tsDCS-concurrent recording of skin conductance, cardiac and respiratory activity (to assess UEs in bodily state). The most frequent participant-reported AEs were sensations of burning, tingling, and itching, although they were largely described as mild; skin redness (experimenter-reported) occurred even more frequently. Importantly, when comparing AEs between active and sham tsDCS via frequentist and Bayesian analysis approaches, the results were largely in favour of no difference between conditions (with the exception of skin redness). A similar picture emerged for most UE metrics, suggesting that tsDCS does not induce changes in bodily state, at least as measured by our autonomic nervous system metrics. We believe that the strategy employed here could serve as a starting point for a systematic AE and UE assessment in clinical populations, longitudinal designs, and when targeting different spinal sites. Taken together, our results contribute to assessing the tolerability and specificity of tsDCS, in order to further the application of this spinal neuromodulation method in health and disease.

## 1 Introduction

The spinal cord serves as a hub for the processing and transmission of neural signals between the body and the brain, essential for motor control, somatosensory processing, and autonomic function (Hochman, 2007). Modulating spinal cord function via invasive stimulation has been employed clinically for decades (Jensen & Brownstone, 2019; Sdrulla et al., 2018), but more recently non-invasive approaches have become feasible as well (Nardone et al., 2015; Taylor et al., 2021). Specifically, transcutaneous spinal direct current stimulation (tsDCS) has emerged as a technique for modulating spinal cord excitability (Albuquerque et al., 2018; Bączyk et al., 2021; Cogiamanian et al., 2012; Priori et al., 2014; Stolbkov & Gerasimenko, 2022). Numerous studies have indicated that tsDCS has a modulatory effect on spinal processing related to somatosensory, nociceptive, and reflex responses (Cogiamanian et al., 2008, 2011; Hubli et al., 2013; Lamy et al., 2012; Truini et al., 2011; Winkler et al., 2010), suggesting that tsDCS could be a useful tool for investigating spinal cord function in health and disease.

Despite a rapidly growing body of tsDCS studies, the field is lacking systematic studies investigating tsDCS adverse effects (AEs; here defined as subjectively reported sensations associated with tsDCS) and unspecific effects (UEs; here defined as concurrently recorded changes in the participants’ physiological bodily state), although such an assessment is important for several reasons. First, it would help to ensure the tolerability of tsDCS by assessing potential risks and discomfort. Second, it would support finding a range of parameter settings that allow for proper blinding, as is of utmost importance especially in clinical settings. Third, being aware of off-target UEs would allow for more informed study design by taking potential confounds into consideration.

Here, we, therefore, comprehensively assessed possible AEs and UEs induced by tsDCS, focusing on four different aims with the first being addressed via a systematic review and the others being addressed via experimental work. First, we performed a systematic keyword search across all published human tsDCS studies to provide an overview of previous work on AE and UE characterization. While such approaches have already been carried out for tDCS (Brunoni et al., 2011; Matsumoto et al., 2017; Nikolin et al., 2018), they are currently lacking for tsDCS. Second, in a preregistered study, we performed a detailed questionnaire-based assessment of AEs, including their spatiotemporal properties as well as blinding success. Third, we investigated UEs via tsDCS-concurrent recordings of several physiological parameters to comprehensively assess possible changes in participants’ bodily state. Importantly, both AEs and UEs were assessed in a within-participant design, allowing us to investigate the effects of different stimulation polarities (anodal, cathodal) compared with sham stimulation. Finally, we aimed to provide evidence for not only the possible existence of AEs and UEs, but also for their possible absence (by complementing frequentist analyses with a Bayesian approach; see, e.g., Keysers et al., 2020), allowing for a rigorous assessment of the tolerability of tsDCS.

## 2 Materials and Methods

### 2.1 Assessing adverse effects (AEs) and unspecific effects (UEs) in previous tsDCS work

To gain an overview of the reporting of AEs and UEs in previous tsDCS studies, we carried out a literature search in the following way. First, following PRISMA guidelines (Moher et al., 2009), we performed a systematic literature search across PubMed, Scopus, Web of Science, and Google Scholar to identify all published human tsDCS studies. For this part, we used the search terms for “study identification” reported in Supplementary Table S1a. Second, after having identified these studies, we conducted 2 full-text screenings within the resulting pool of 76 articles: 1 for adverse effects (AEs; search terms listed in Supplementary Table S1b) and 1 for unspecific effects (UEs; search terms listed in Supplementary Table S1c). Based on this process, 45 studies reporting AEs and 11 studies mentioning UEs were identified (which were overlapping). Additionally, we explored whether studies reporting positive outcomes in our AE search incorporated questionnaires for AE assessment by examining occurrences of the terms “assessment” and “questionnaire”.

### 2.2 Participants

Twenty healthy volunteers (10 females, mean age: 30.1 years, range: 20–40 years) participated in this study after providing written informed consent. The study was approved by the ethics committee at the Medical Faculty of Leipzig University.

The sample size was determined based on an a-priori power analysis, as specified in a preregistration on the Open Science Framework (https://osf.io/d9tyv). While the power analysis resulted in a suggested sample size of 40 participants, we also incorporated an optional stopping rule (coming into play after data acquisition and interim analysis of 20 participants) whereby a stable Bayes Factor (>3 or <0.3) would lead to an early stop of data acquisition to make most efficient use of resources.

### 2.3 Experimental design

This study is part of a larger preregistered tsDCS project (ClinicalTrials.gov ID: NCT05711498). We used a randomized, double-blind, sham-controlled, within-participant design. All participants took part in three sessions, each of which featured a different stimulation condition (anodal, cathodal, sham), with the order being balanced across participants. In order to ensure that participants were aware of the experimental design, the Participant Information Sheet informed them about receiving three different stimulation conditions.

Double-blinding was assured via the following procedure. The experimental randomization (i.e., order of anodal, cathodal, or sham stimulation across visits) was created (via a random number generator) by a researcher who was not involved in data acquisition or tsDCS administration. The experimenters acquiring the data were blind to stimulation conditions, and participants were likewise unaware of which condition they received in each session. A dedicated tsDCS operator, who was aware of the assigned stimulation condition (via the above-mentioned list), only administered tsDCS, but was not involved in data acquisition. To maintain blinding, the tsDCS operator remained in the laboratory for 20 minutes in both active and sham sessions before leaving. To conclude, neither the participants nor the investigator performing the experiment was aware of the stimulation conditions—only the tsDCS operator was aware of stimulation condition but did not have any involvement in data acquisition or participant interaction.

Sessions were separated by at least 1 week (preventing possible carry-over effects from previous sessions), occurred at the same time of day (minimizing effects of diurnal variation) and participants did not take part in other neurostimulation studies during the study (preventing confounding effects).

### 2.4 Transcutaneous spinal direct current stimulation (tsDCS)

tsDCS was carried out using a direct current stimulator (DC-Stimulator Plus, neuroConn, Ilmenau, Germany) with electrodes placed over the thoracic spinal cord (spinous process of the 12th thoracic vertebra) and the right shoulder (suprascapular region). The location of the spinal electrode was chosen as a compromise: on the one hand, we aimed to follow previous studies that placed electrodes at a more rostral site (e.g., 10th thoracic vertebra, Cogiamanian et al., 2011), and on the other hand, we aimed to take into account more recent modelling work suggesting that a more caudal placement (e.g., second lumbar vertebra, Fernandes et al., 2018) is superior for targeting the relevant spinal segments. The target areas were cleaned with alcohol wipes to remove surface grease from skin and thus lower the impedance. We used rectangular rubber electrodes of 7 x 5 cm size (neuroConn, Ilmenau, Germany) covered with electrode paste (Ten20 Conductive Paste, Weaver and Company, Aurora, USA). Stimulation consisted of a fade-in of 15 seconds, a plateau of 20 minutes (with stimulation at 2.5 mA either anodally or cathodally) and a fade out of 15 seconds, with tsDCS polarity referring to the electrode placed over the spinal cord. The chosen stimulation parameters (intensity of 2.5 mA and duration of 20 minutes) are the most commonly employed parameters (see also Table 1). A stimulation of this amplitude in combination with the chosen electrodes results in a current density of 0.071 mA/cm^2^, well below thermal and histological limits for current density (see also Antal et al., 2017). Sham stimulation followed the anodal montage with 15-second fade in and fade out, but only 45 seconds of plateau stimulation at 2.5 mA. Throughout the experiment, participants were seated in a comfortable, relaxed position, with back supported, knees slightly flexed, and muscles relaxed throughout the session.

**Table 1.** Adverse effects reported in previous work.

							Reported adverse effects in relation to tsDCS						
First author	Year	Journal	Sample size	Stimulation polarity (A = anodal, C = cathodal, S = sham)	Stimulation intensity (mA)/duration (minutes)	AE questionnaire	itching/itchy	tingling	burning	redness	irritation	sensation	discomfort
*Healthy volunteer studies*													
Albuquerque	2018a	PLoS One	12	A, C, S	2.5/20	no	x	x					
Awosika	2019	Brain Stimulation	43	A, S	2.5/20	yes	x		x				x
Berry	2017	PLoS One	12	A, S	2.5/15	no	x	x				x	
Bocci	2015a	Journal of Neuroscience Methods	10	A, C, S	2/20	no		x	x				
Bocci	2015b	Neuromodulation	10	A, C, S	2/20	no		x				x	
Bocci	2015c	Journal of Neurophysiology	14	A, C	2.5/20	no	x	x	x				
Clark	2022	Frontiers in Aging Neuroscience	23	A, S	2.5/30	yes	x	x	x				
Cogiamanian	2008	Clinical Neurophysiology	12	A, C, S	2.5/15	no	x	x	x				
Cogiamanian	2011	Pain	11	A, C, S	2/15	no	x	x	x				
Donnelly	2021	Scientific Reports	23	A, C, S	2/20	no		x					
Fava de Lima^a,d^	2022	PLoS One	17	A, C, S	5/20	yes	x	x	x				
Jadczak	2019	Frontiers in Physiology	31	A, C, S	2.5/15	no	x			x			
Lamy	2012	Journal of Neurophysiology	22	A, C, S	2.5/15	no	x	x					
Lenoir	2018	Neuroscience	15	A, S	2.5/20	no	x	x					
Meyer-Frießem	2015	Neuroscience Letters	24	A, S	2.5/15	no		x					
Murray	2018	Scientific Reports	22	A, C, S	4/30	no	x	x					
Murray & Knikou	2019	Experimental Brain Research	10	C	2–2.7/10	yes	x	x	x	x	x		
Niérat	2014	Journal of Neuroscience	22	A, C, S	2.5/15	no	x	x					
Pereira	2018	Clinical Neurophysiology	14	A, C, S	2.5/15	no	x		x				
Perrotta	2016	Clinical Neurophysiology	10	A, C, S	2/15	no		x					
Powell ^a^	2019	NeuroRehabilitation	9	A, C	2, 2.5, 3 /20	no							
Ruggiero	2019	Neuropsychologia	37	A, S	2/20	no	x	x	x				
Schweizer	2017a	Clinical Neurophysiology	26	A, C, S	2.5/15	no	x	x					
Schweizer	2017b	Brain Connectivity	20	A, C, S	2.5/20	yes		x					
Thordstein	2020	Journal of Clinical Neuroscience	19	A	2.5/20	no	x			x			
Truini	2011	European Journal of Pain	17	A, C	2.5/20	no		x	x				
Winkler	2010	Clinical Neurophysiology	10	A, C, S	2.5/15	no	x	x					
*Patient studies*													
Alhassani	2017	Hong Kong Physiotherapy Journal	9	A	1/20	no	x	x	x			x	
Ardolino	2021	The Journal of Spinal Cord Medicine	11	A, S	2/20	yes	x	x					
Awosika	2020	Brain Communications	30	A, S	2.5/30	yes	x	x	x			x	
Benussi ^b^	2021	Brain	61	C, S	2/20	no		x					
Berra	2019	Frontiers in Human Neuroscience	33	A, S	2/20	no		x					
Choi	2019	Spinal Cord	10	A, S	2/20	no		x					
Guidetti	2021	Frontiers in Neurology	16	A, S	2.5/20	yes						x	
Hawkins	2022	Spinal Cord	8	A, S	2.5/30	no			x		x		
Heide	2014	Brain Stimulation	34	A, C, S	2.5/15	no		x				x	
Hubli	2013	Clinical Neurophysiology	34	A, C, S	2.5/20	no	x	x					
Lamy ^c^	2021	Movement Disorders	16	A, C, S	2.5/20	no							
Marangolo	2020	Brain Research	16	A, S	2/20	yes	x	x	x			x	
Paget-Blanc	2019	Bioelectronic Medicine	26	C, S	4/20	no						x	
Picelli	2015	Restorative Neurology and Neuroscience	30	A, C, S	2.5/20	no						x	
Pisano	2020	Journal of Alzheimer’s Disease	16	A, S	2/20	no		x					
Pisano	2021	Behavioural Brain Research	10	A, S	2/20	no		x					
Rahin	2023	Brain Sciences	21	A, S	2.5/20	no		x	x	x			
Wang	2020	Sleep Medicine	50	A, S	2/20	no		x					
Zeng	2020	Frontiers in Neuroscience	50	A, S	2/20	no		x					

This table provides details for all studies in which our systematic keyword search for tsDCS AEs returned hits.

a Reported blisters (due to used gel or contact with the stimulation electrodes).

b The anode was placed on the scalp over the cerebellum area (2 cm under the inion).

c Reported one case of mild headache.

d Reported heating, pain, and duration of electrical stimulation, mentioned temporary mild erythema under the electrodes.

### 2.5 Data acquisition

#### 2.5.1 Recording AEs via structured questionnaire

Based on a proposal for a tDCS questionnaire (Brunoni et al., 2011), we developed a structured tsDCS questionnaire (Supplementary Fig. S1) that allowed us to systematically record (i) potential AE symptoms, (ii) the relation of reported AEs to tsDCS, (iii) participants’ guesses about the authenticity of tsDCS (active or sham; Question 1), (iv) participants’ guesses about the direction of tsDCS (inhibitory or excitatory; Question 2), and (v) the onset time, duration, and location of reported AEs (Questions 3–5). The symptom report part (including Questions 3–5) was administered immediately after tsDCS and the questions related to blinding (i.e., Questions 1–2) were answered at the end of a session, that is, after all reflex recordings were finished. For each session, only one questionnaire was completed by the participant (i.e., one questionnaire for each of anodal, cathodal, and sham stimulation). However, participants were explicitly instructed that they could provide additional information regarding the location of any adverse effects, including sensations relating to active (back) or reference (shoulder) electrode.

#### 2.5.2 Recording UEs via autonomic nervous system measures

During tsDCS, three different physiological signals relevant to autonomic nervous system activation (skin conductance, electrocardiographic, and respiratory signals) were acquired from recommended recording sites at 2500 Hz using a BrainAmp ExG system (Brain Products GmbH, Gilching, Germany). Skin conductance was recorded by two electrodes placed on the thenar and hypothenar eminence of the right hand, electrocardiographic data were recorded with one electrode placed at the left lower costal arch and referenced to a right sub-clavicular electrode, and respiratory data were recorded via a breathing belt around the lower rib cage.

### 2.6 Data processing

#### 2.6.1 AEs and blinding success

Participants’ ratings of each symptom were scored on a severity scale from 1 to 4 (1: absent, 2: mild, 3: moderate, 4: severe). As these ratings were also used to compute an “Aggregate Symptom Score” (by summing up the ratings across all symptoms), we adjusted them to a scale of 0 to 3, with 0 signifying the absence of AEs in the respective session. Participants’ ratings regarding the relation of symptoms to tsDCS were scored on a scale from 1 to 4 (1: not related, 2: remotely related, 3: probably related, 4: definitely related).

Participants’ answers to questions 1 (“Do you think that today was an active stimulation or a sham stimulation condition?”) and 2 (“If active, do you think it was inhibitory or excitatory stimulation?”) were used to assess blinding success, using the following classification: “Active + Inhibitory” was classified as “Anodal”, “Active + Excitatory” was classified as “Cathodal”, and “Sham” was classified as “Sham”. The terms “inhibitory” and “excitatory” were used solely with respect to sensory spinal processing, where anodal tsDCS has an inhibitory effect (e.g., Cogiamanian et al., 2011) and cathodal tsDCS has an excitatory effect (e.g., Cogiamanian et al., 2008). Questions 3 and 4 captured the onset and duration of reported AE symptoms (where participants’ responses were binned into six temporal categories) and question 5 assessed the spatial distribution of AEs (where participants’ responses were binned into four spatial categories).

#### 2.6.2 UEs

All data processing for UEs was carried out using Python 3.9. The summary measures of tsDCS-concurrent physiological signals were extracted for the whole time period of tsDCS and for quarters of that time period.

##### 2.6.2.1 Skin conductance fluctuations (SCF)

Data were down-sampled to 100 Hz and filtered via a bidirectional first-order Butterworth bandpass (passband: 0.0159 Hz to 5 Hz). Spontaneous SCF were quantified via an area under the curve approach, whereby we interpolated over all local minima of the skin conductance time series and determined the area between this baseline signal and the actual time series (Bach et al., 2010).

##### 2.6.2.2 Electrocardiographic (ECG) activity

R-peaks were automatically detected using a Pan–Tompkins Algorithm (Pan & Tompkins, 1985) implemented in the Python package py-ecg-detectors (https://github.com/berndporr/py-ecg-detectors) and manually corrected. Heart rate (HR) was determined by averaging the heart beats per minute and heart rate variability (HRV) was calculated as the root mean square of successive differences.

##### 2.6.2.3 Respiratory activity

The time points that mark the beginning of a new breathing cycle were automatically detected by determining the signal minima (representing maximum inhalation). Breathing rate (BR) was determined as the number of breaths per minute and breathing rate variability (BRV) was assessed as the standard deviation of the interval between consecutive breaths.

### 2.7 Statistical analysis

As specified in a preregistered analysis plan, we mostly employed one-tailed tests and established statistical significance at a level of p < 0.05. In addition to frequentist tests, we also employed a Bayesian approach by comparing the evidence for the null model against alternative models using Bayes Factors (BF), which allowed us to determine evidence for the presence or absence of an effect (Keysers et al., 2020). All analyses were carried out in JASP (JASP Team, 2023; version 0.17.3.0; using default uninformed priors), separately for anodal vs sham and cathodal vs sham (i.e., no direct polarity contrasts, following a preregistered analysis plan).

#### 2.7.1 AEs and blinding success

To assess condition differences in AEs, each item of the tsDCS AE symptom report was analyzed separately using a Wilcoxon sign-rank test and the same analysis was carried out on the Aggregate Symptom Score. For the per-item tests, we also indicate whether a significant test result survived Bonferroni correction for the number of items. The participants’ guesses regarding the stimulation condition were analyzed with a McNemar test (not available in Bayesian implementation). The Aggregate Symptom Scores in correctly vs. incorrectly guessing participants were compared with a Mann–Whitney U test.

#### 2.7.2 UEs

The analysis of physiological data was complicated by the fact that in some cases, participants had inadvertently not been instructed not to talk and move during tsDCS administration, leading to abnormal signal fluctuations in these participants’ autonomic measures and the exclusion of several participants’ data (Supplementary Table S2; three participants had to be excluded completely and six participants had to have one session excluded). We compared SCF, HR, HRV, BR, and BRV values between anodal and sham as well as between cathodal and sham. Overall effects (assessing the entire stimulation window) were investigated using paired-samples t-tests and time-dependent effects (quarters of the stimulation window, about 5 minutes each) were investigated using a 2 x 4 repeated-measures ANOVA, comparing anodal vs sham and cathodal vs sham separately (necessary due to the uneven distribution of missing data mentioned above).

The BF reported for the paired-samples t-tests (BF_10_) indicate the likelihood ratio of the observed data given the alternative hypothesis that the two measures are different in comparison with the null hypothesis that the values are equal. For example, a BF of 3 means that the data are three times more likely to be observed under the alternative than the null and a BF of 1/3 means that the data are three times more likely to be observed under the null than the alternative (conventionally described as providing moderate evidence for the presence or absence of an effect; Keysers et al., 2020).

For the repeated-measures ANOVA, we were interested in the interaction effect of condition and time and report two BF. BF_10_ indicates the likelihood ratio of the observed data given the alternative model that includes the two main factors and the interaction in comparison with the null model that does not contain these elements. BF_incl_ indicates the likelihood ratio of the observed data given models that include the interaction term in comparison with the models that do not include the interaction term.

### 2.8 Terminology

In this study, we employ the terms “adverse effect” and “adverse event” as defined in Chapter 19 of the Cochrane Handbook for Systematic Reviews of Interventions (Peryer et al., 2024). Specifically, an “adverse event” is defined as “an unfavourable or harmful outcome that occurs during, or after, the use of a drug or other intervention, but is not necessarily caused by it” and an “adverse effect” is defined as “an adverse event for which the causal relation between the intervention and the event is at least a reasonable possibility”. Note that the use of this definition does not mean to deny that there are many intricacies in the use of these terms (Aronson, 2023). The employed tsDCS questionnaire (Supplementary Fig. S1) furthermore allowed for a cautious differentiation between adverse effects and adverse events based on participant reports, as participants indicated for each symptom how likely they judged it to be caused by tsDCS. Please note that our use of the term “adverse effect” differs from what is suggested in the most recent guidelines for transcranial electric stimulation (Antal et al., 2026), where solely the term “adverse event” is employed. In the future, we will also make use of this term and urge other researchers to do likewise when building on the work reported here.

## 3 Results

### 3.1 Assessing AEs and UEs in previous work

We identified 76 human tsDCS studies, of which 17 did not report any AE search terms (Supplementary Table S3), 14 mentioned at least 1 search term, but did not observe AEs (Supplementary Table S4), and 45 reported AEs (Table 1). Among the latter, tingling was reported in 36 studies, itching in 24 studies, and burning in 16 studies, with lesser reports of skin-related irritations/sensations, skin redness, and discomfort. Across all included studies, no serious adverse events were reported or described (Supplementary Table S5). An AE assessment based on questionnaires was only carried out in 10 studies and the level of reported details was rather limited (Table 2). A keyword search for UE reporting revealed hits in 11 studies (Albuquerque et al., 2018; Ardolino et al., 2021; Awosika et al., 2020; Clark et al., 2022; Guidetti et al., 2021; Hodaj et al., 2023; Marangolo et al., 2020; Murray & Knikou, 2019; Niérat et al., 2014; Pisano et al., 2021; Ruggiero et al., 2019), but with the exception of 1 study (Niérat et al., 2014; who assessed polarity-dependent changes in spontaneous breathing patterns), none obtained tsDCS-concurrent recordings without potential confounds, that is, the relevant measures were primarily used as an indicator to ensure adequate task performance.

**Table 2.** Questionnaire-based adverse effects assessment in previous work.

First author	Year	Journal	Sample size	Stimulation polarity (A = anodal, C = cathodal, S = sham)	Questionnaire for tsDCS adverse effects	Verbatim report of questionnaire results
*Healthy volunteer studies*						
Awosika	2019	Brain Stimulation	43	A, S	Tolerability, activity, and safety questionnaire	“Participants’ reports on verbal 0/10 scales indicated the following. In the anodal and sham groups, general discomfort was 1.27 (range 0–5) and 0.90 (range 0–4), perception of pain was 0.18 (range 0–2) and 0.24 (range 0–3), sensation of burning under the electrode was 0.50 (range 0–2), and 0.43 (range 0–5), and itching under the electrode was 0.63 (range 0–2) and 0.76 (range 0–5), respectively. No skin irritation or burns occurred. Thus, tsDCS was overall well tolerated.”
Clark	2022	Frontiers in Aging Neuroscience	23	A, S	“Participants used an 11-point rating scale where 0 represents “none” and 10 represents “strongest/worse possible.” For tsDCS, the following items were rated: tingling, itching, burning, pain, fatigue, nervousness, headache, muscle spasms, mood change, urinary urgency, abdominal/pelvic sensations, and sweating.”	“no adverse effects”… “For tsDCS there were reports of very mild tingling/burning sensation at the electrode sites (average rating less than 1 out of 10). All other potential side effects of tsDCS were negligible or completely absent.”
Fava de Lima	2022	PLoS One	17	A, C, S	At the end of each experimental session, participants were asked to fill out an assessment form. The subjects had to rate on a five-level scale the duration of the electrical stimulation and on a four-level scale the perceived levels of itching, pain, burning, heating, and tingling from the electrical stimulation.	“No statistically significant differences were obtained between different electrical stimulation protocols indicating satisfactory experimental blinding. No major adverse effects were reported. All participants presented temporary mild erythema under the electrodes positioned on the back and on the iliac crests. Two subjects developed small blisters on the skin in the region in contact with the stimulation electrodes which healed in a few days. One subject presented hypersensitivity to the electrical stimulation, cancelling the participation in the experiment.”
Murray & Knikou	2019	Experimental Brain Research	10	A, C, S	A tsDCS questionnaire was administered “to establish the presence of any adverse effects”, but no further specification was provided.	“Following tsDCS, the major complaint was skin redness or irritation which subsided within a few hours, followed by reports of tingling, burning or itchy sensations mainly during the ramp-up and down phase of stimulation.”
Schweizer	2017b	Brain Connectivity	20	A, C, S	“After each session, subjects completed a questionnaire to assess any pain associated with tsDCS as well as their guess as to which polarity of tsDCS they had received.” There was no further specification of the questionnaire.	“No adverse effects from the tsDCS electrodes […] were reported.”
*Patient studies*						
Ardolino	2021	The Journal of Spinal Cord Medicine	11	A, S	tsDCS adverse effects questionnaire (Brunoni et al., 2011)	“In general, the experimental procedures were well tolerated by all subjects and only a few subjects reported an occasionally slight tingling or itching sensation beneath the electrodes. No difference was distinguishable between the “real” or “sham” stimulation nor between polarities in relation to sensations caused by stimulation (e.g., itching, tingling, or auditory perception).”
Awosika	2020	Brain Communications	30	A, S	Tolerability, activity, and safety Questionnaire	The authors report mean, median, and standard deviation of each questionnaire item for anodal and sham stimulation in their Table 2, as well as p-values for the stimulation condition comparison (none of which were significant)
Guidetti	2021	Frontiers in Neurology	16	A, S	tsDCS adverse effects questionnaire (Brunoni et al., 2011)	“No adverse effects were reported”
Hodaj^a^	2023	Brain Communications	36	A, S	Comfort rating questionnaire (Palm et al., 2014)	“No adverse effects were reported during or following any of the three interventions.”
Marangolo	2020	Brain Research	16	A, S	Sensation questionnaire (Fertonani et al., 2010)	“No adverse sensations were reported. Participants did not recognize which condition they were in and they did not detect a difference in sensations between stimulation conditions (Paired sample t-tests: itchiness: t_(15)_ = -0.19, p = 0.85; pain: t_(15)_ = -0.37, p = 0.72; burning: t_(15)_ = 0.24, p = 0.82; warmth/heat: t_(15)_ = -0.27, p = 0.79; pinching: t_(15)_ = -0.37, p = 0.72; fatigue: t_(15)_ = 0.17, p = 0.87).”

This table provides details for all studies that used a questionnaire to assess possible AEs of tsDCS.

a The study by Hodaj and colleagues is not listed in Table 1, but in Supplementary Table S4, since these authors did not report AEs.

### 3.2 Assessing AEs

#### 3.2.1 Symptom reports

**Fig. 1.**
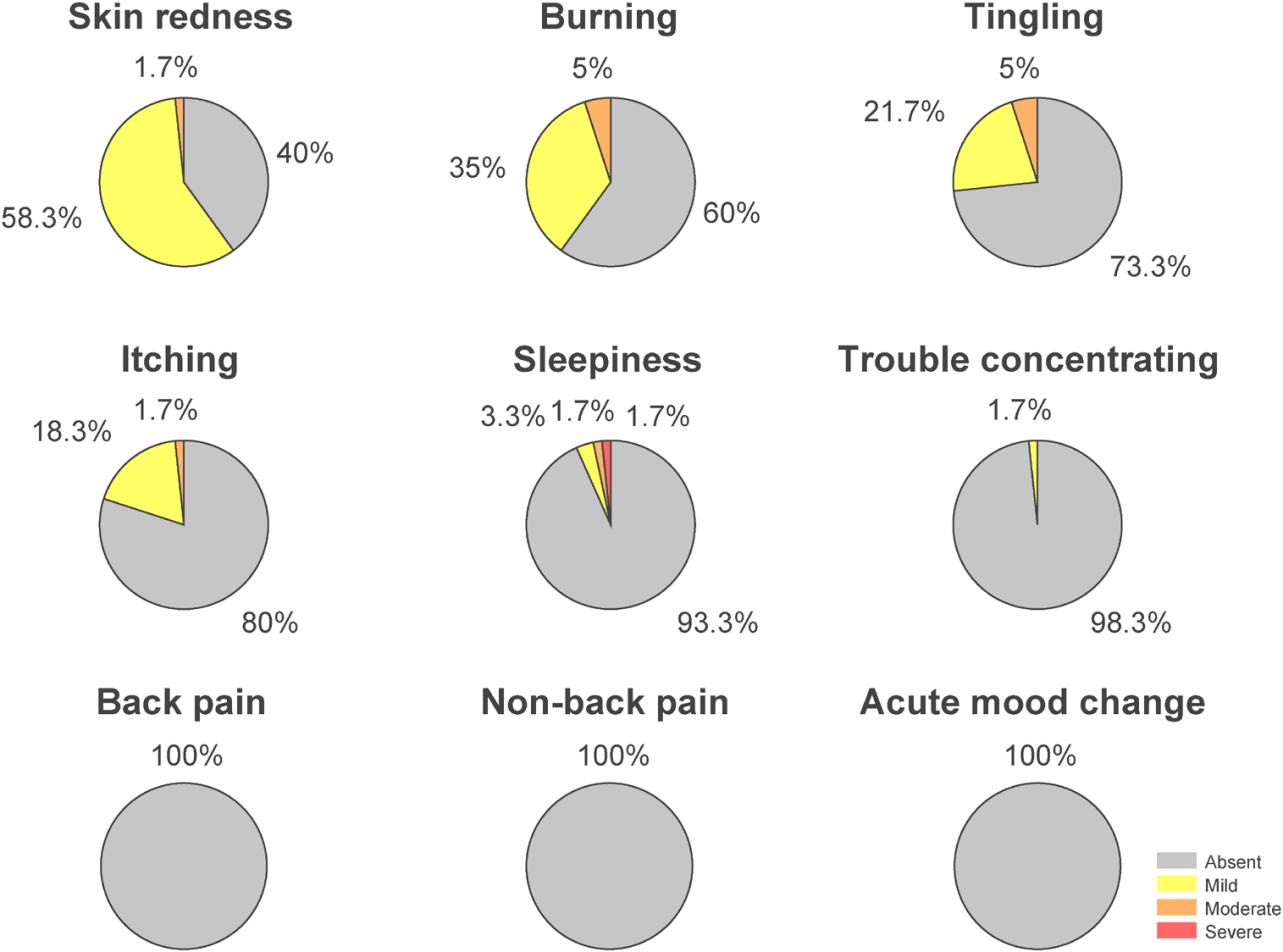
Adverse effects reports. The occurrence and severity of AEs are based on all 60 sessions, with colors representing the severity of the reported adverse effects (see legend).

Turning to our own study, when aggregating data across all conditions in terms of participant-reported symptoms (Fig. 1), burning (40.0%), tingling (26.7%), and itching (20.0%) were the predominant AEs (mostly of mild severity), with skin redness (60%) being reported by the experimenter and having the highest occurrence overall and other AEs being virtually non-existent across all 60 sessions.

**Fig. 2.**
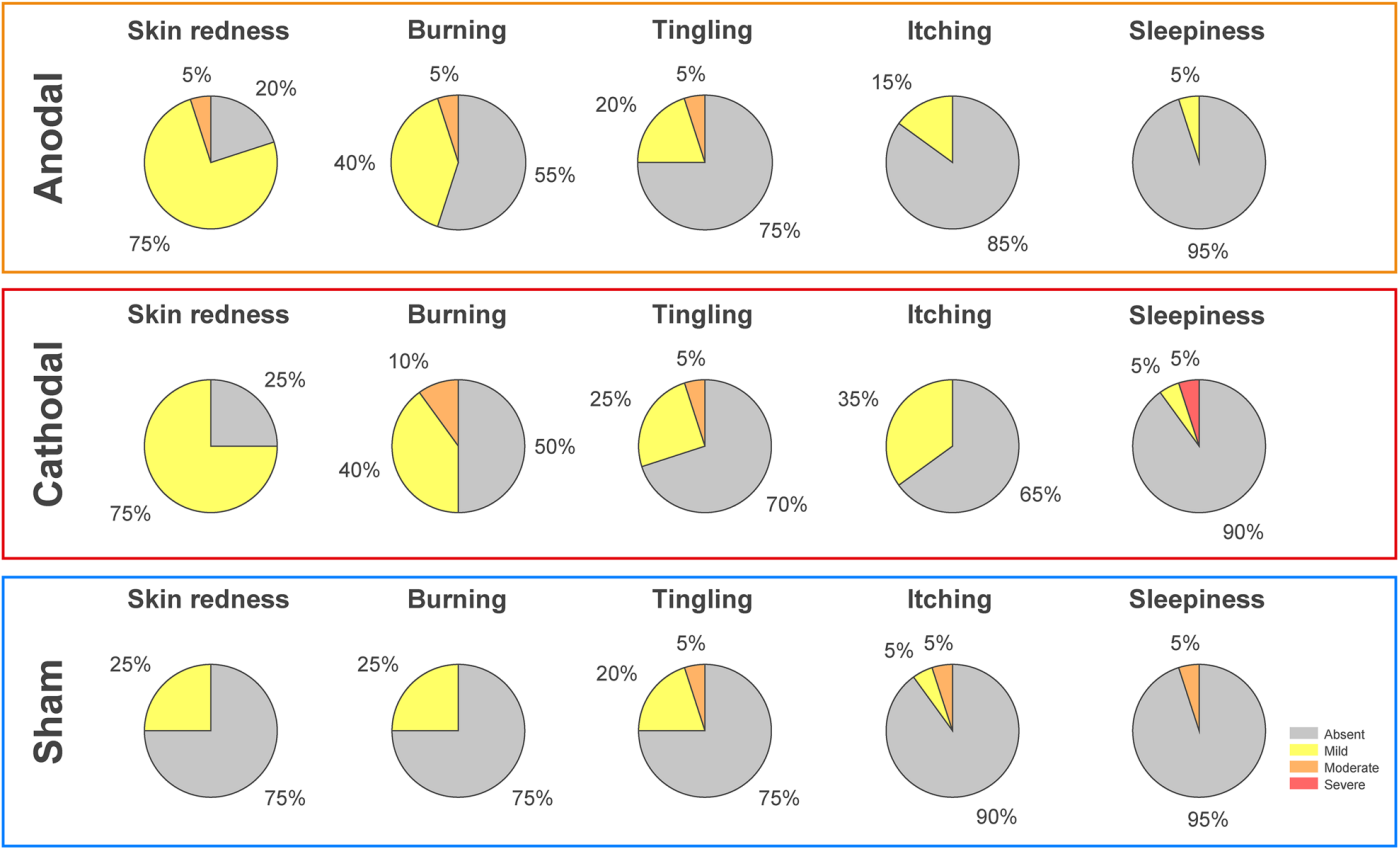
Adverse effects reported across conditions. The occurrence and severity of AEs are depicted dependent on condition (anodal, cathodal, sham; each based on 20 participants), with colors representing the severity of the reported adverse effects (see legend). Note that back pain, non-back pain, acute mood change, and trouble concentrating are not displayed here due to the absence of reports (except for one report of trouble concentrating in the cathodal group).

None of the participant-reported symptoms showed significant differences between conditions and only the experimenter-reported item of skin redness exhibited strong evidence for a difference between the active and sham conditions (Fig. 2; Table 3). From a Bayesian perspective, the results clearly favoured the null-hypothesis of no condition differences in participant-reported symptoms over the alternative hypothesis (7/8 BF < 1, 5/8 BF < 1/3, and 0/8 BF > 3). As for the Aggregate Symptom Score, no significant differences were observed for anodal vs sham (with the BF being supportive of a null effect), but for cathodal vs sham a marginally significant effect was observed, though not paralleled by the BF analysis, indicating inconclusive evidence.

**Table 3.** Statistical comparison of AEs.

Comparisons	Anodal *vs* Sham		Cathodal *vs* Sham	
	Wilcoxon signed-rank test		Wilcoxon signed-rank test	
Reported AEs	p value	BF_10_	p value	BF_10_
Skin redness	0.002*	42.07	0.002*	22.45
Tingling	1	0.27	1	0.29
Itching	1	0.27	0.29	0.48
Burning	0.19	0.58	0.06	1.45
Sleepiness	1	0.30	0.75	0.31
Back pain	—	—	—	—
Non-back pain	—	—	—	—
Trouble concentrating	—	—	—	—
Acute mood change	—	—	—	—
	Anodal *vs* Sham		Cathodal *vs* Sham	
Comparisons	p value	BF_10_	p value	BF_10_
Symptom Score	0.43	0.30	**0.042**	1.53

Note that the symptom score does not include skin redness, that is, only aggregates participant-reported symptoms.

Bold font is used to indicate significant results at an uncorrected level and an asterisk is used to indicate significant results after Bonferroni correction for the number of tests performed. 1. For back pain, non-back pain, trouble concentrating, and acute mood change, the reported frequencies were extremely low (almost zero, with only 5% in the cathodal group for trouble concentrating). This led to within-group variances of 0, rendering these comparisons unfeasible, and consequently, the results are indicated as “—”. 2. Bayes factor indication: BF > 3: moderate evidence for condition difference; BF < 0.33: moderate evidence for absence of condition difference; 0.33 < BF < 3: insufficient evidence for or against either effect.

Regarding the reported relation between AEs and tsDCS, skin redness, tingling, itching, and burning were reported as highly associated with tsDCS, while sparsely reported symptoms exhibited a much weaker reported relationship with tsDCS (Supplementary Fig. S2).

#### 3.2.2 Assessing participant blinding

When assessing participants’ assumptions regarding the type of stimulation they had received (“Active” or “Sham”), 5% indicated they had received 0 of 3 active sessions, 20% thought 1 of 3 were active, 50% indicated that 2 of 3 were active, and 25% believed 3 of 3 were active. Upon assessing participants’ reports regarding the specific stimulation type they had received, 5% of participants had entirely incorrect answers, 55% had one correct answer, 35% had two correct answers, and only 5% had entirely correct answers. When testing whether participants were able to correctly guess the stimulation condition, we observed no significant effects (anodal vs sham: p = 0.74; cathodal vs sham: p = 0.62). Based on the Aggregate Symptom Score, we also explored whether participants’ subjective symptom experiences were related to the accuracy of their guesses, but found no evidence for this: all p > 0.4 and all BF < 0.6.

#### 3.2.3 Assessing temporo-spatial AE properties

**Fig. 3.**
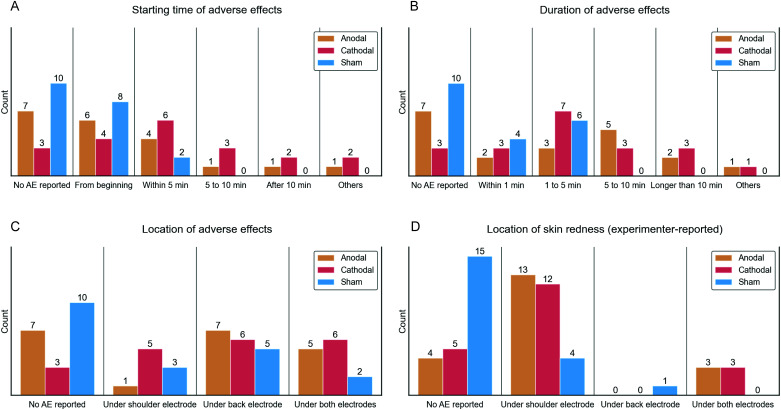
Temporal and spatial adverse effects patterns. Depicted reports of onset time (A), duration (B), location (C) of participant-reported AEs, and location (D) of experimenter-reported item across different stimulation conditions. Note that the category “Others” was introduced as some participants reported differences in onset times and durations of AEs for electrodes, thus preventing an assignment to a unique category. Bars represent absolute number of reports among the sample of 20 participants.

The reported AEs exhibited distinct patterns in terms of onset time, duration, and location across the different stimulation conditions (Fig. 3). In the sham condition, no AEs were observed in half of the participants and the onset of the reported AEs mostly occurred during the tsDCS initiation phase and all within the first 5 minutes (Fig. 3A). In the active conditions, AE onset showed a clear shift towards later onset times compared with the sham condition. With respect to the duration of AEs (Fig. 3B), all reported AEs for sham stimulation occurred within the initial 5 minutes, whereas reported AEs for active stimulation conditions had a much longer duration: 40% of participants reported a duration of longer than 5 minutes under anodal stimulation and 35% did so under cathodal stimulation. Most AEs were reported to occur at the back electrode site in both active and sham stimulations. This was followed by reports of occurrence under both electrodes, yet here more prominently in active compared with sham conditions (Fig. 3C): only 10% of participants reported this under sham, but 25% under anodal and 30% under cathodal. Experimenter-reported skin redness was notably absent in the majority of participants (75%) during sham stimulation, contrasting with active stimulation, where it predominantly occurred at the shoulder electrode site in 65% of participants under anodal stimulation and 60% under cathodal stimulation, compared with 20% under sham stimulation (Fig. 3D).

### 3.3 Assessment of unspecific effects (UEs)

**Fig. 4.**
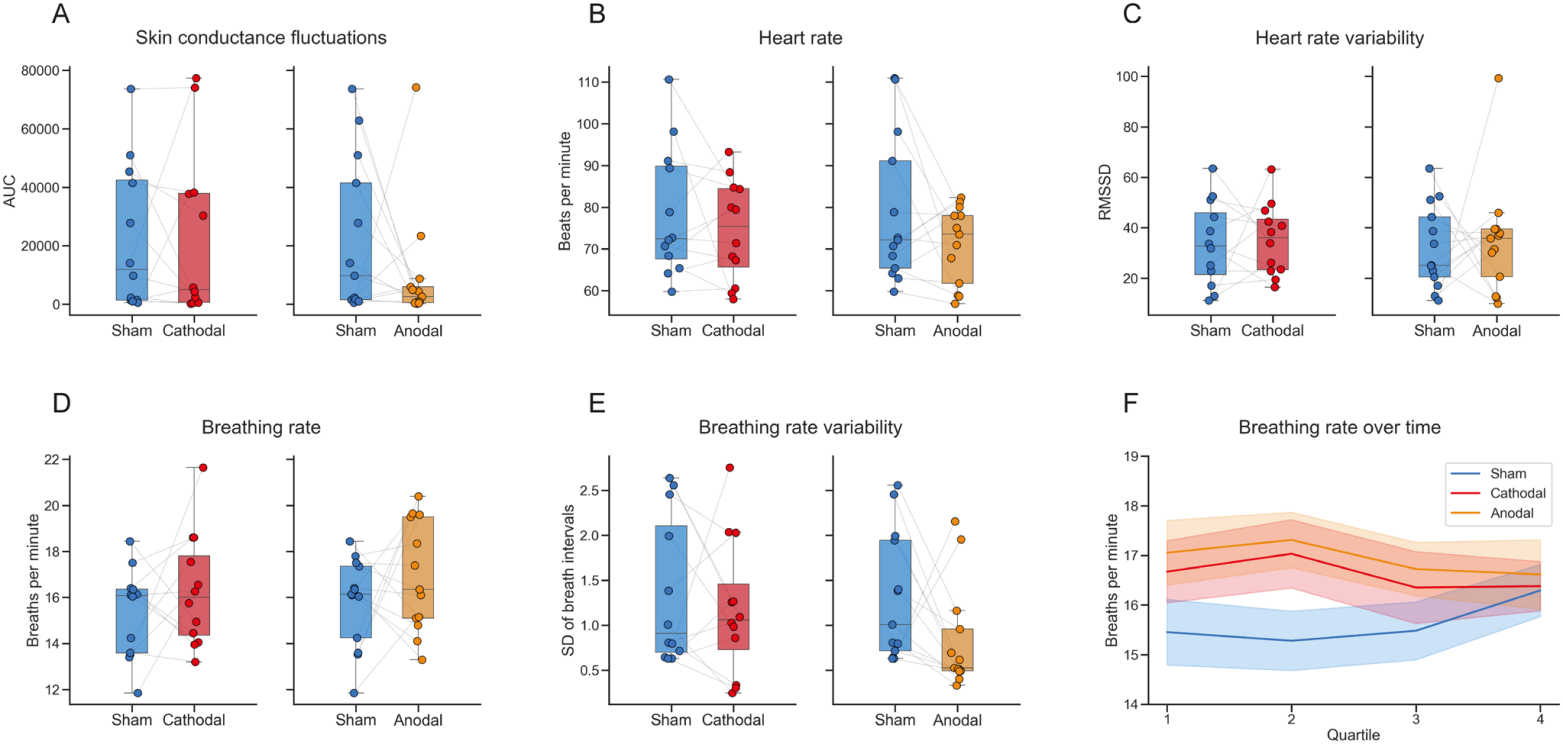
Unspecific effects assessment via autonomic responses in different stimulation conditions. Comparison of spontaneous skin conductance fluctuations (A), heart rate (B), heart rate variability (C), breathing rate (D), and breathing rate variability (E) between cathodal and sham as well as between anodal and sham conditions, respectively. Note that the sham group does not always consist of the same data points as participants were excluded from specific sessions due to excessive noise (see description in Methods section). (F) Depiction of group-level means and standard error of the mean underlying the significant time-by-condition interaction in breathing rate.

Participant-specific and group-level scores of the tsDCS-concurrent physiological measures are depicted in Figure 4. Out of the 10 statistical comparisons, none showed significant differences and all BF were below 1, with four instances providing moderate evidence for an absence of condition differences (BF < 1/3; Table 4). To assess whether tsDCS-induced unspecific effects might have developed differentially over time, we tested for a time-by-condition interaction, but in 8 out of 10 statistical comparisons, we did not observe significant interactions and in 7 of those, BF provided moderate-to-strong evidence against an interaction effect (Table 5). Only for breathing rate did we observe a significant interaction, but the BF were equivocal and further investigation showed that this interaction was largely driven by a change of breathing rate in the sham condition (Fig. 4F).

**Table 4.** Statistical comparison of UEs.

Comparisons	Anodal *vs* Sham		Cathodal *vs* Sham	
	Paired samples t-test		Paired samples t-test	
Autonomic responses	p value	BF_10_	p value	BF_10_
Skin conductance fluctuations	0.26	0.49	0.98	0.29
Heart rate	0.15	0.73	0.40	0.40
Heart rate variability	0.80	0.29	0.80	0.30
Breathing rate	0.27	0.48	0.32	0.45
Breathing rate variability	0.11	0.87	0.57	0.33

Bayes factor indication: BF > 3: moderate evidence for condition difference; BF < 0.33: moderate evidence for absence of condition difference; 0.33 < BF < 3: insufficient evidence for or against either effect.

**Table 5.** Statistical comparison of UEs: 5-minute intervals interaction effect.

Comparisons	Anodal *vs* Sham			Cathodal *vs* Sham		
	rm-ANOVA condition × time			rm-ANOVA condition × time		
Autonomic responses	p value	BF_incl_	BF_10_	p value	BF_incl_	BF_10_
Skin conductance fluctuations	0.21	0.16	0.08	0.26^a^	0.08	0.04
Heart rate	0.74^a^	0.02	0.01	0.27^a^	0.10	0.05
Heart rate variability	0.85	0.05	0.03	0.63^a^	0.05	0.02
Breathing rate	**0.02**	0.77	0.37	**0.02**	2.76	1.50
Breathing rate variability	0.23	0.11	0.06	0.09^a^	0.95	0.42

Bold font is used to indicate significant results at an uncorrected level. BF_incl_ indicates the change from prior to posterior inclusion odds (referring to the sum of the prior or posterior probabilities of all models that include the effect). BF_10_ indicates the comparison of our interaction model (including the two main effects) with a null model (containing only subject and random slopes). Bayes factor indication: BF > 3: moderate evidence for the tested effect; BF < 0.33: moderate evidence against the tested effect; 0.33 < BF < 3: insufficient evidence for or against either effect.

a Greenhouse–Geisser correction used.

## 4 Discussion

Here, we investigated AEs and UEs associated with tsDCS, by first performing a review of the tsDCS literature in this regard and then empirically assessing AEs and UEs in a preregistered study via a structured questionnaire and tsDCS-concurrent physiological recordings, respectively.

### 4.1 Adverse effects (AEs) of tsDCS

To comprehensively assess tsDCS AEs in a structured way, we developed a questionnaire based on an existing tDCS template (Brunoni et al., 2011) and employed this in a randomized, within-participant, double-blind design involving 20 participants (who underwent anodal, cathodal, and sham tsDCS). This allowed us to provide detailed descriptions of overall AE reports as well as condition differences using Frequentist and Bayesian statistics, including spatio-temporal AE aspects and blinding success. To our knowledge, this combination of factors goes far beyond what has previously been carried out in the tsDCS literature: out of 76 human tsDCS studies, only 10 (Ardolino et al., 2021; Awosika et al., 2019, 2020; Clark et al., 2022; Fava de Lima et al., 2022; Guidetti et al., 2021; Hodaj et al., 2023; Marangolo et al., 2020; Murray & Knikou, 2019; Schweizer et al., 2017) employed structured questionnaires, with only 3 of these statistically comparing effects under active and sham conditions (Ardolino et al., 2021; Awosika et al., 2019; Marangolo et al., 2020) and none investigating spatio-temporal aspects. Our study thus provides a starting point for a systematic and comprehensive assessment of tsDCS AEs, and we believe that the tsDCS community might benefit from a standardized and psychometrically evaluated questionnaire.

Our findings revealed predominantly mild AEs, mostly consisting of skin-related sensations at the electrode sites, such as burning, tingling, and itching. Participants rated these events as strongly related to tsDCS and hence we use the term “adverse effects” to describe them (in contrast to “adverse event”, which could be a coincidental occurrence, such as sleepiness). While this is in line with prior reports in the tDCS (Matsumoto et al., 2017) as well as the tsDCS literature (Cogiamanian et al., 2008, 2011; Murray & Knikou, 2019; Pisano et al., 2021; Ruggiero et al., 2019), we went beyond these previous reports by conducting both frequentist and Bayesian comparisons between active and sham conditions for each AE. In none of the comparisons did we observe a significant difference on any item, and complementary Bayesian analyses provided moderate evidence for an absence of condition differences in half of these comparisons. There were no reports of painful sensations or acute mood changes, which is in line with reporting in the tsDCS literature, where—across almost 80 studies—head pain (Awosika et al., 2020) and musculoskeletal pain (Hawkins et al., 2022) were each only reported once; across all included studies, no serious adverse events were reported. We furthermore observed only very few reports of sleepiness and trouble concentrating (which were rated as unlikely to be related to tsDCS). Taken together, this suggests that—from the perspective of participant reports—tsDCS is a well-tolerated and safe technique, consistent with previous reports on the adverse effects of tDCS (Nitsche et al., 2003).

We also asked participants about the onset, duration, and location of experienced AEs and observed that under active stimulation conditions, reported AEs lasted longer (i.e., no participants reported AEs to last longer than 5 minutes during sham, but 35% did so during cathodal stimulation and 40% during anodal stimulation) and there were slightly more reports of AEs under both electrodes in the active conditions (25% of participants for anodal and 30% for cathodal stimulation) than the sham condition (10%). While previous tsDCS studies mostly focused on the presence or absence of AEs (Ardolino et al., 2021; Clark et al., 2022) and did not report temporal features of AEs, we believe that a spatio-temporal characterization of AEs as carried out here is important for allowing to design an appropriate tsDCS control condition that ensures adequate blinding.

### 4.2 Participant and experimenter blinding

Apart from AEs, we also investigated participants’ assumptions regarding the type of stimulation they received. While 50% of participants correctly reported that two sessions were active—suggesting their attentiveness to instructions (Rabipour et al., 2018), considering that this information was provided at experiment start and also upon questionnaire administration—only 5% were correct in assigning all three conditions, suggesting good blinding performance. While such a lack of correct condition assignment is in line with previous tsDCS studies (Albuquerque et al., 2018; Berry et al., 2017; Lamy et al., 2012; Lenoir et al., 2018; Meyer-Frießem et al., 2015; Niérat et al., 2014; Schweizer et al., 2017; Truini et al., 2011; Winkler et al., 2010), we went beyond this simple dichotomy and also explored whether participants’ accuracy in reporting the stimulation condition was associated with differences in reported AEs: reassuringly, also here we did not observe significant differences, suggesting that adequate blinding on the participant side occurs even with a tsDCS intensity of 2.5 mA as carried out here.

It is important to consider, however, that the experimenter-assessed item of skin redness clearly differentiated between active and sham conditions, potentially leading to experimenter unblinding in the worst case (O’Connell et al., 2012). Contrary to our observations (where skin redness was the most prominent AE), skin redness was only reported four times in the tsDCS literature (Jadczak et al., 2019; Murray & Knikou, 2019; Rahin et al., 2023; Thordstein et al., 2020), without reports of significant differences between active and sham conditions as observed here, thus deserving further study. Another aspect to consider is how skin redness evolves over time, as participants could potentially unblind themselves regarding active vs sham stimulation by looking at their back/shoulder after the experiment. Here, it is important to point out that we only assessed skin redness once, that is, at the end of an experimental session, without follow-up at later time points. A further consideration for future research could be the use of pre-set encrypted stimulation programs on the DC stimulator, such that not even the presence of a tsDCS operator would be necessary.

Overall, we believe that it is prudent to formally assess blinding success regularly in tsDCS studies as well as investigate other approaches to sham stimulation, such as different electrode placement or expectation manipulation via de-facto masking (O’Connell et al., 2012; Wallace et al., 2016).

### 4.3 Unspecific Effects (UEs) of tsDCS

We also assessed whether active compared with sham tsDCS induces UEs in bodily state and observed consistently non-significant results as well as Bayes Factors mostly indicating an absence of condition differences. This pattern of results suggests that active thoracolumbar tsDCS does not modulate vital functions such as heart rate or breathing rate, which is reassuring from a safety perspective. Despite our findings, we believe that further research is necessary to replicate and extend these results, considering that our systematic review indicated that this field is virtually untouched: one study (Hodaj et al., 2023) investigated longitudinal post-tsDCS changes in skin conductance (though in patients where an autonomic dysfunction is part of the pathology) and another study (Niérat et al., 2014) assessed changes in spontaneous breathing as well as skin conductance and heart rate (though the latter two not in a polarity-dependent or sham-controlled manner).

The absence of effects on autonomic function observed here is also noteworthy when considering the spatial proximity of our stimulation site (12th thoracic vertebra) to some of the autonomic outflow pathways. The sympathetic nervous system originates from the T1 to L3 levels of the spinal cord (Darby, 2014; McCorry, 2007), with a focus on T1 to T5 for upper limb and cardiac innervation. Modelling studies exploring the E-field of thoracolumbar tsDCS (Bastos et al., 2016; Fernandes et al., 2018; Kuck et al., 2017) suggest that such thoracic segments could be affected by our type of tsDCS (though future thoracolumbar tsDCS studies might also consider recording skin conductance from the foot, as the innervating segments will be closer to the active electrode). Conversely, the phrenic motoneurons innervating respiratory muscles are located in the spinal segments C3–C5 (Verin et al., 2011), which should not be affected by our type of tsDCS. Taken together, we believe that the tsDCS community should routinely record autonomic signals during experiments, as these are easy to obtain and would offer important insights into tsDCS’s specificity and tolerability.

### 4.4 Limitations and future directions

Several limitations of our study are worth mentioning. First, our AE and UE assessment occurred in young healthy volunteers and thus has limited generalizability to other populations. Second, a more comprehensive exploration of bodily states (including metrics such as blood pressure and cortisol levels) would offer a more holistic understanding of the off-target impact of tsDCS. Third, our focus on the acute effects of tsDCS does not allow any inferences on the cumulative effects of repeated tsDCS sessions, as would be relevant clinically. Fourth, our results only pertain to thoracolumbar tsDCS and it is thus essential to carry out similar studies for cervical tsDCS (which might have different UEs, such as respiratory effects; see also Niérat et al., 2014). Along the same lines, it would be worth to explore AEs and UEs also when using different electrode montages at the thoracolumbar level, such as dual-electrode spinal placement (Kuck et al., 2017; Fernandes et al., 2018; Pereira et al., 2022), which could lead to different effects, as could different stimulation intensities and durations. Fifth, our use of the terms “inhibitory” and “excitatory” solely pertains to the effects of tsDCS on nociceptive read-outs as employed in this study (e.g., withdrawal reflexes and pain ratings) and is not meant to suggest that we can directly infer such tsDCS effects on neuronal processing. Finally, our results suggest that maintaining experimenter and participant blinding requires considerable attention in future studies and possibly also more sensitive assessments of blinding success than the here-employed “end-of-study guess” (Turner et al., 2021).

## 5 Conclusion

Our investigation into the AEs and UEs of tsDCS demonstrates that tsDCS is a safe and well-tolerated technique, whose AE profile is primarily characterized by mild skin-related effects. Our UE findings furthermore indicate that tsDCS does not cause alterations in core autonomic measures and could thus be expected to exert rather specific neural effects. Taken together, our study provides substantial contributions to the understanding of adverse effects and specificity of tsDCS as well as participant blinding. It should be followed up by similar approaches in clinical populations and longitudinal studies to unlock the full potential of tsDCS for understanding and modulating spinal cord function in health and disease.

## Supplementary Material

Supplementary Material

## Data Availability

The underlying data are openly available (https://osf.io/f7spw/), as is all analysis code (https://github.com/eippertlab/tsdcs-sideeffects).
