# Supplementary material for: Assessing adverse effects and unspecific effects of transcutaneous spinal direct current stimulation (tsDCS)
Source: Imaging Neurosci (Camb). 2026 Jul 14;4:IMAG.a.1292. doi: 10.1162/IMAG.a.1292 (PMC13370751; doi:10.1162/IMAG.a.1292)
Supplement: Supplementary Material [file IMAG.a.1292_supp.pdf]

Supplementary Material

Assessing adverse effects and unspecific effects of transcutaneous spinal direct current stimulation (tsDCS)

Hongyan Zhao, Ulrike Horn, Melanie Freund, Anna Bujanow, Christopher Gundlach, Gesa Hartwigsen, Falk Eippert

tsDCS Adverse Effects Questionnaire

Possible adverse effects	Did you experience any of the listed adverse effects? Please enter a number (1: absent, 2: mild, 3: moderate, 4: severe).	If present, do you think this is related to tsDCS? Please enter a number (1: not related, 2: remotely related, 3: probably related, 4: definitely related).	Additional notes
Back pain			
Non-back pain			
Tingling			
Itching			
Burning sensation			
Skin redness			
Sleepiness			
Trouble concentrating			
Acute mood change			
Others (please specify)			

Additional questions

1. Do you think that today was an active stimulation or a sham stimulation condition?

   ☐ Active ☐ Sham

2. If active, do you think it was inhibitory or excitatory stimulation?

   ☐ Inhibitory ☐ Excitatory

3. If you had any of the above-described symptoms, when did they start?

   When: _____________ (when did you firstly feel any sensation, i.e., how many seconds/minutes after the stimulation started)

4. If you had any of the above-described symptoms, for how long did they last?

   Duration: __________ seconds / minutes

5. If you had any of the above-described sensory symptoms, where did you feel them?

   Location: __________

Supplementary Figure 1. tsDCS Adverse Effects Questionnaire. The questionnaire, developed based on a proposed template for tDCS (Brunoni et al. 2011), captures potential adverse effect symptoms, their relation to tsDCS, participant guesses regarding the tsDCS condition, and details on adverse effects' onset, duration, and location.

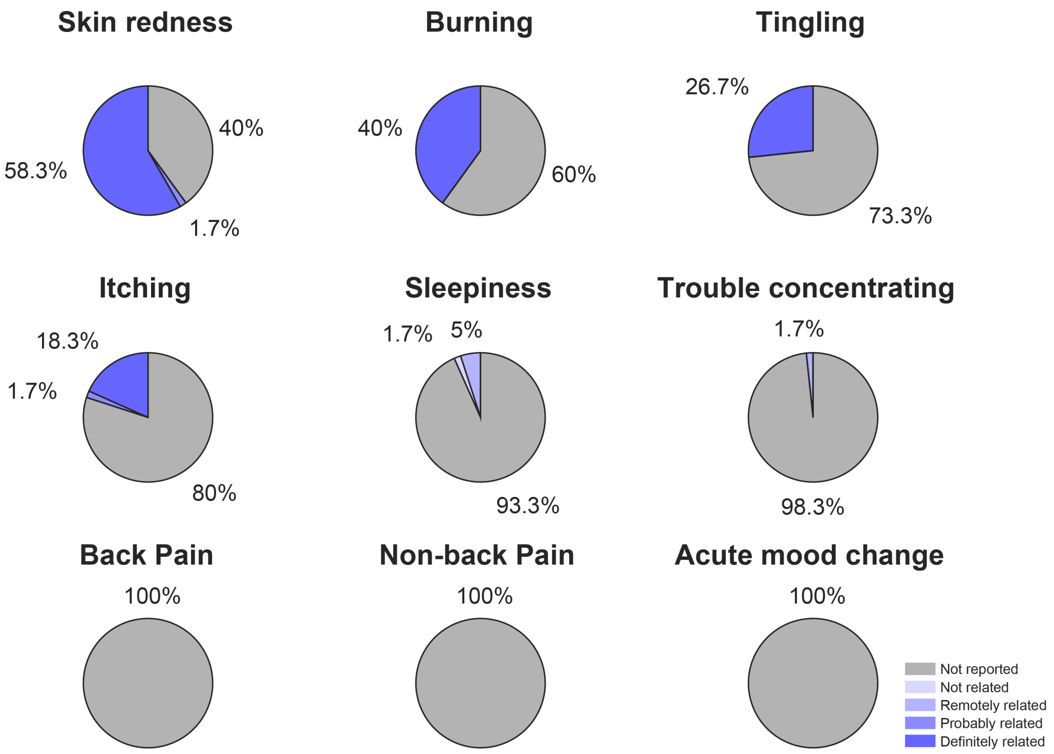

Supplementary Figure 2. **Relation reports of adverse effects with tsDCS.** The relation of reported AEs is based on all 60 sessions, with colors representing the relation degree (see legend).

Supplementary Table 1. Search terms for a) identifying studies, b) identifying the reporting of AEs and c) UEs.

Aims	Search terms
a	"transcutaneous spinal direct current stimulation", "trans cutaneous spinal direct current stimulation", "trans-cutaneous spinal direct current stimulation", "transspinal direct current stimulation", "trans spinal direct current stimulation", "trans-spinal direct current stimulation", "tsDCS" (all in English)
b	"adverse", "irritation", "burning", "itching/itchy", "tingling", "discomfort", "sensation", "redness", "side effect"
c	"respiration", "respiratory", "breath", "breathing", "heart(-)rate", "heart(-)period", "cardiac", "cardiovascular", "electrocardiography", "electrocardiogram", "ECG", "skin conductance", "SCR", "electrodermal", "sudomotor", "galvanic", "EDA", "GSR"

Supplementary Table 2. Details on participant exclusion for UE analyses.

Participant number	Session number	Reason
01	2	talking
08	2	coughing
09	all sessions	excessive movement and talking
10	3	movement
13	all sessions	talking
14	all sessions	talking
16	3	talking
17	3	talking
19	1	movement

Supplementary Table 3. List of all studies in which our keyword search for tsDCS AEs did not return any hits.

First author	Year published	Journal
Healthy volunteer studies		
Bocci	2014a	Neuroscience Letters
Ciccone	2021	The Journal of Strength and Conditioning Research
Donges	2017a	Experimental Physiology
Donges	2017b	PLoS One
Gibson	2019	Neuroscience Letters
Koseki	2023	Frontiers in Neuroscience
Lamy	2013a	Journal of Neurophysiology
Sasada*	2017	Neuroscience Letters
Therkildsen*	2021	Experimental Brain Research
Yamaguchi	2020	Physiological Reports
Patient studies		
Abualait	2020	Saudi Medical Journal
Benussi	2018	Neurology
Benussi	2019	Brain Stimulation
Gogeascoechea	2020	Frontiers in Neurology
Kobayashi	2022	2022 International Conference on Rehabilitation Robotics (ICORR)
Zhang*	2021	IEEE Transactions on Neural Systems and Rehabilitation Engineering

*Sasada et al., 2017 and Therkildsen et al., 2021 returned keyword "discomfort", and Zhang et al., 2021 returned "adverse effect" after search, but none of those were related to tsDCS.

Supplementary Table 4. List of all studies in which our keyword search for tsDCS AEs did return hits, but where no detailed AEs were reported.

First author	Year published	Journal
Healthy volunteer studies		
Bettmann	2020	Scientific Reports
Kamali	2021	Scientific Reports
Kamali	2023	Scientific Reports
Kuck	2018	Frontiers in Neuroscience
Lim	2011	NeuroReport
Patient studies		
Adeel	2022a	Journal of the Formosan Medical Association
Hodaj	2023	Brain Communications
Lin*	2022	Experimental Brain Research
Marangolo	2017	Frontiers in Neurology
Naro	2022	Brain Sciences
Picelli	2018	Restorative Neurology and Neuroscience
Picelli	2019	Restorative Neurology and Neuroscience
Powell	2016	NeuroRehabilitation
Powell	2018b	NeuroRehabilitation

*In Lin et al., 2022, an absence of adverse effects was reported, with examples of absence mentioned (such as itching and burning sensation).

Supplementary Table 5. Summary of whether studies reported similar adverse events (AEs) under sham stimulation and whether serious adverse events (SAEs) were described.

**First author**	**Year**	**Journal**	**Any similar AEs reported in sham group**	**Any descriptions of serious adverse events**
Healthy volunteer studies				
Albuquerque	2018a	PLoS One	n.a.	no
Awosika	2019	Brain Stimulation	Similar mild sensations (discomfort, pain, burning, itching) were reported in both active and sham sessions	no
Berry	2017	PLoS One	n.a.	no
Bocci	2015a	Journal of Neuroscience Methods	n.a.	no
Bocci	2015b	Neuromodulation	n.a.	no
Bocci	2015c	Journal of Neurophysiology	No sham	no
Clark	2022	Frontiers in Aging Neuroscience	n.a.	no
Cogiamanian	2008	Clinical Neurophysiology	Itching sensation similar to active stimulation	no
Cogiamanian	2011	Pain	Itching sensation similar to active stimulation	no
Donnelly	2021	Scientific Reports	Itching sensation similar to active stimulation	no
Fava de Lima	2022	PLoS One	Itching, Pain, Burning, Heating, Tingling	"No major adverse effects were reported"
Jadczak	2019	Frontiers in Physiology	Itching sensation similar to active stimulation	no
Lamy	2012	Journal of Neurophysiology	Itching sensation similar to active stimulation	no
Lenoir	2018	Neuroscience	Moderate tingling and itching sensation	no
Meyer-Friessem	2015	Neuroscience Letters	Tingling	no
Murray	2018	Scientific Reports	n.a.	no
Murray	2019a	Experimental Brain Research	n.a.	no
Nierat	2014	Journal of Neuroscience	n.a.	no
Pereira	2018	Clinical Neurophysiology	n.a.	no
Perrotta	2016	Clinical Neurophysiology	n.a.	no
Powell	2018a	NeuroRehabilitation	No sham	no
Ruggiero	2019	Neuropsychologia	Itching	no
Schweizer	2017a	Clinical Neurophysiology	n.a.	no
Schweizer	2017b	Brain Connectivity	Itching sensation similar to active stimulation	no
Thordstein	2020a	Journal of Clinical Neuroscience	No sham	no
Truini	2011	European Journal of Pain	n.a.	no
Winkler	2010	Clinical Neurophysiology	n.a.	no
Patient studies				
Alhassani	2017	Hong Kong Physiotherapy Journal	No sham	no

Ardolino	2021	The Journal of Spinal Cord Medicine	n.a.	no
Awosika	2020	Brain Communications	Headache, neck pain or pain, tingling, itching, burning	“There were no serious adverse events in the study”
Benussi	2021	Brain	Tingling	no
Berra	2019	Frontiers in Human Neuroscience	n.a.	no
Choi	2019	Spinal Cord	Itching sensation similar to active stimulation	no
Guidetti	2021	Frontiers in Neurology	Skin sensations are indistinguishable from anodal tsDCS	no
Hawkins	2022	Spinal Cord	n.a.	no
Heide	2014	Brain Stimulation	Itching sensation similar to active stimulation	no
Hubli	2013	Clinical Neurophysiology	Tingling	no
Lamy	2021	Movement Disorders	n.a.	no
Marangolo	2020	Brain Research	Tingling, itching	no
Paget-Blanc	2019	Bioelectronic Medicine	n.a.	no
Picelli	2015	Restorative Neurology and Neuroscience	n.a.	no
Pisano	2020	Journal of Alzheimer's Disease	Tingling	no
Pisano	2021	Behavioural Brain Research	Tingling	no
Rahin	2023	Brain Sciences	During real tsDCS, 90% of participants felt mild tingling or burning at the electrodes (mostly at stimulation onset); for sham, 75% experienced the same.	no
Wang	2020	Sleep Medicine	Tingling	no
Zeng	2020	Frontiers in Neuroscience	Tingling	no

n.a.: Reporting of AEs was not differentiated between active and sham stimulation.
